# Effect of Seclusion on Mental Health Status in Hospitalized Psychiatric Populations: A Trial Emulation using Observational Data

**DOI:** 10.1177/01632787231164489

**Published:** 2023-03-10

**Authors:** Stéphanie Baggio, Stefan Kaiser, Alexandre Wullschleger

**Affiliations:** 1 Institute of Primary Health Care (BIHAM), 27230 University of Bern, Bern, Switzerland; 2 Division of Prison Health, 27230 Geneva University Hospitals and University of Geneva, Geneva, Switzerland; 3 Adult Psychiatry Division, Department of Psychiatry, 27230 Geneva University Hospitals, Geneva, Switzerland

**Keywords:** coercion, ethics, inpatient psychiatry, seclusion

## Abstract

The use of coercive practices, i.e., interventions against a person’s will, is controversial. Recent observational studies highlighted their potential detrimental effects on patients’ mental health, but this topic remains understudied. This study investigated the effect of a common coercive practice, seclusion (i.e., being locked in a closed room), on mental health using a trial emulation of observational data to allow causal inference. We used data from 1200 psychiatric inpatients, classified as being either secluded or non-secluded during their hospital stay. Inverse probability of treatment weighting was used to emulate the random assignment to the intervention. The primary outcome was the Health of the Nations Outcome Scales (HoNOS). The secondary outcome was the first item of the HoNOS, which focuses on overactive, aggressive, disruptive, or agitated behavior. Both outcomes were assessed at hospital discharge. There was a significant effect of seclusion with increases in both total HoNOS score (*p* = .002) and item 1 of the HoNOS (*p* = .01). Seclusion may have a negative causal effect of patients’ mental health status and should therefore be avoided in mental health care settings. Training efforts should raise the awareness of the medical staff about potential adverse effects instead of therapeutic benefits.

Coercive practices, i.e., the use of interventions against a person’s will, are commonly used in mental health care settings. Coercive measures include involuntary admission, seclusion (i.e., being locked in a closed room), physical restraint, and forced treatment. They are mostly used to manage aggressive behaviors or in life-threatening situations that cannot be managed otherwise (Newton-Howes, 2013).

The present study focused on seclusion, which is commonly used in adult inpatient psychiatry. Seclusion is most used to prevent self-harm and harm of others because of aggressive behavior. Seclusion was the most used coercive measure in the setting where the study took place (Chieze et al., 2021a, 2021b). Coercive measures are controversial because they may violate several principles, even if they are unfortunately sometimes inevitable.

First, coercion is a threat to human rights, as it overrules individuals’ will and preferences (Gooding et al., 2020). Coercion violates the central guiding principle of autonomy, which allows patients to make their own decisions about treatment (Sugiura et al., 2020). Consequently, there is a growing international policy momentum to reduce the use of coercive measures in psychiatry (see for example the initiative Fostering and Strengthening Approaches to Reducing Coercion in European Mental Health Services, https://fostren.eu) and recent research discussed prevention and reduction initiatives (Barbui et al., 2021; Gooding et al., 2020).

Second, there is a growing concern that such practices have a negative effect on patients, for both physical and mental health (Chieze et al., 2019; Kersting et al., 2019). In a recent systematic review focusing on physical harm and death, Kersting et al. (2019) showed that seclusion was associated with receiving less care and an increase in self-harm. This study nonetheless concluded that seclusion remained understudied. In another systematic review investigating associations between seclusion and psychological outcomes, Chieze et al. (2019) suggested that seclusion had deleterious consequences, including the development of post-traumatic symptoms, feelings of punishment, distress, and increased length of hospital stay.

Third, there is a lack of evidence-based evaluation of the clinical consequences of the use of seclusion. This has already been pointed out in the early 2000s (Finke, 2001), but conclusions are still relevant nowadays (Chieze et al., 2019). Few high-quality studies investigated the effect of coercive measures on patients’ mental health outcomes. To our knowledge, three randomized controlled trials (RCTs) investigated the effect of coercive measures in psychiatric populations (Bergk et al., 2011; Huf et al., 2012; Vaaler et al., 2005), but none compared seclusion to a control condition without seclusion and limiting the risk of bias has been difficult in these studies (Chieze et al., 2019). Prospective observational studies investigating the effect of seclusion had severe limitations. It included cross-sectional designs, selection bias, lack of power, and lack of adequate confounding adjustment (Soininen et al., 2013; Whitecross et al., 2013).

Despites these important ethical controversies, coercive measures are still used in psychiatry, with potential large variations between countries and settings (Hotzy et al., 2018; Välimäki et al., 2019). There are potential favorable attitudes of some health care professionals toward the use of coercive measures (e.g., therapeutic effect of coercive measures) (Chieze et al., 2019; Doedens et al., 2019; Van Der Merwe et al., 2013).

Further studies with robust methods are thus needed to provide empirical evidence on the effects of seclusion. Most importantly, a better understanding of the consequences of seclusion on mental health outcomes is needed. This is especially true after the beginning of the SARS-CoV-2 pandemic, as seclusion has been elected as a way to quarantine SARS-CoV-2 cases (Lodhi & Marett, 2020).

To fill in these research gaps, we emulated a trial using observational data to investigate the effect of seclusion on mental health status. A trial emulation is a technique that mimics a RCT using observational data. It is used when RCT are not feasible or ethical and allows causal inference (Hernán & Robins, 2016). The primary outcome was the Health of the Nations Outcome Scales (HoNOS) at hospital discharge. As seclusion is a way to deal with aggression (Newton-Howes, 2013), we considered the first item of the HoNOS, which focuses on overactive, aggressive, disruptive, or agitated behavior, as a secondary outcome.

## Methods

### Study Design

The “target trial” is the RCT we would have designed if it was feasible and ethically acceptable. In our case, the target trial would randomly assign participants to either use of seclusion or nonuse of seclusion during hospitalization, at hospital admission. An overview of the target trial is provided in the first column of Table 1. We used observational data from medical files of the Geneva University Hospitals, Geneva, Switzerland, to emulate a target trial of the effect of seclusion on mental health status of adult patients hospitalized in psychiatric wards (see second column of Table 1). Participants were followed-up from admission to discharge.

**Table 1.** Description of the Target Trial Emulation

Component	Target Trial	Emulated Trial
Aim	To estimate the relative effect of seclusion on mental health status in patients hospitalized in adult psychiatry	Same
Design	Prospective open lab two parallel arm superiority randomized trial	Retrospective cohort study
Eligibility criteria	*Inclusion criteria*	*Inclusion criteria*
	Age ≥18	Same
	Being hospitalized in the adult psychiatric ward of the geneva university hospitals	Same
	Being hospitalized between Mar 2020 and Dec 2020	Same
	*Exclusion criteria*	*Exclusion criteria*
	Did not consent to participate	Refusal to reuse of data for research purposes
Treatment strategies	1) Use of seclusion during hospitalization	Patients are assigned to the group 1) or 2) if they were/were not secluded during their hospitalization
	2) No use of seclusion during hospitalization	
Assignment procedures	Participants randomly assigned to either strategy at hospital admission and aware of the strategy they are assigned to	Randomization is emulated via adjustment for all hypothesized confounding factors identified a priori
Follow-up	*Start:* Time of treatment assignment (admission)	*Start:* Hospital admission
	*Stop:* Hospital discharge	*Stop:* Same
Outcomes	*Primary outcome:* HoNOS at discharge *Secondary outcomes:* Item 1 HoNOS at discharge	Same
Causal contrasts	ITT effect	Observational analogue of PP effect
	PP effect	
Analysis plan	*ITT:* Compare means between randomized groups	*PP:* Same as PP analysis
	*PP:* Compare means between groups receiving/not receiving the treatment, with patients who deviate from protocol being censored and use of inverse probability weighting to adjust for baseline covariates and attrition	

*Note*. HoNOS: Health of the Nations Outcome Scales, ITT: Intention to treat, PP: per protocol.

### Study Setting

The present trial is based on data collected for a larger study investigating the effects of the SARS-CoV-2 pandemic on hospitalization rates and use of coercive measures. Anonymized routine data were collected from the hospital’s electronic files. The Geneva’s cantonal ethics committee approved the study protocol (no. 2021-00263).

In the Geneva University Hospitals, the 14 inpatient wards of the department of psychiatry admit patients aged 18 or more, having severe mental illnesses. Most wards apply an open-door policy. There were around 1900 admissions in 2020 with a mean duration of stay of 24 days. We excluded three inpatient units that did not apply seclusion.

Following Swiss federal law, in the Geneva University Hospitals the use of seclusion is limited to the following situations: (1) imminent risk of aggressive behavior towards others, (2) behavior with a severe disruption of the ward community (putting others at risk), (3) exceptionally to prevent absconding with major risk of harm for self or others. Among these situations, imminent risk of aggressive behavior is the most frequent. Seclusion is only allowed when no other alternative is available to sufficiently reduce the risks. Acute suicide risk is a contraindication for the use of seclusion.

### Eligibility Criteria

Patients were eligible for study participation if they did not decline reuse of their data for research purposes, were aged 18 or more, and were admitted in the adult and geriatric psychiatric wards of the Geneva University Hospitals between March (week 12) and December 2020 (week 52).

### Exposure/Treatment

Participants were classified as being either secluded or non-secluded during their hospital stay. Seclusion was defined as being locked in a room in case of endangerment of others, risk or absconding with endangerment of others or oneself, or severe disorganization that cannot be managed otherwise. Seclusion was coded as present (if used at least once during the hospital stay) or absent, without consideration of the duration or number of seclusion episodes.

## Outcomes

*Primary outcome.* The total HoNOS score at discharge was the primary study outcome (score 0–48) (Wing et al., 1998).

*Secondary outcome*. The first item of the HoNOS at discharge, which rates symptoms related to overactive, aggressive, disruptive, or agitated behaviors, was used as a secondary outcome (score 0–4).

### Confounding Factors

Important confounding factors were included in the study. A previous systematic review identified age, gender, ethnicity, psychiatric diagnoses, severity of symptoms, and psychiatric admission history as predicting factors of the use of coercive measures (Beames & Onwumere, 2022). Other studies reported that being single was also a predictive factor (Chieze et al., 2021a, 2021b).

*Sociodemographic variables*. Age, gender, nationality (Swiss versus other), and civil status (recoded as married or registered partnership versus single, divorced, or widower) were recorded.

*Clinical variables*. Previous hospitalizations in psychiatry (yes/no), unvoluntary admission (yes/no), psychiatric ward (adult versus geriatrics), duration of hospitalization (less than 3 weeks versus 3 weeks or more), and HoNOS at admission were recorded. Primary psychiatric disorders were also collected, defined according to ICD-10 (F0-F9) (WHO, 2004). As some disorders were rare in the sample, a principal component analysis was conducted to reduce the number of dimensions. The analysis suggested two categories of disorders: Schizophrenia, bipolar disorder, and personality disorders versus other disorders (dementia, mood disorders, anxiety disorders, intellectual disabilities, substance use disorders, and other disorders). The first category was described as a risk factor of seclusion in previous studies (Beames & Onwumere, 2022; Chieze et al., 2021a, 2021b).

### Statistical Analyses

As this project was a sub-study of a larger project, no sample size was computed a priori. We computed a sensitivity power analysis to assess the minimum effect size the study could detect. With *n* = 290 in the secluded group, *n* = 910 in the non-secluded group, alpha = .05, power = .80, and a two-tailed independent t-test, the effect size was d = .19. Therefore, our study could identify small effect sizes.

We first computed preliminary statistics for the whole sample and for secluded versus non-secluded participants. Descriptive statistics were performed using percentages or means. Comparisons between groups with simple mixed-effect logistic regressions, as participants could have multiple hospital stays.

Then, to emulate the random assignment of the target trial and assess the average causal effect of seclusion on the outcomes, we used inverse probability (IP) of treatment weighting. The goal of IP weighting is to create a pseudo-population in which the treatment is not associated with identified confounders (Hernán, 2022). Stabilized IP weights were used. For this purpose, we first fitted a logistic regression model for the probability of being secluded with all potential confounders included as covariates (the ten sociodemographic and clinical variables described above, the HoNOS score at baseline and item 1 of the HoNOS at baseline). Fitted values were used as the denominator. Second, we fitted a saturated logistic model for the probability of being secluded without any covariate. These fitted values were used as the numerator to compute IP weights, so the probability of being assigned to a treatment strategy did not depend on the confounders. As there were missing values for the HoNOS at discharge, we also used stabilized IP weighting to account for attrition. The denominator was derived by fitting a logistic regression model for the probability of being not censored with all covariates, including seclusion. The numerator was derived by fitting a logistic regression model for the probability of being not censored with seclusion. The final IP weights were a multiplication of these two IP weights, adjusting for both confounding and attrition bias.

For both outcomes, we computed a linear regression model predicting the total HoNOS score/item 1 of the HoNOS at discharge with the treatment strategy (being or not secluded), controlling for covariates and using IP weighting for confounding and attrition bias (Benkeser et al., 2021; Hernán, 2022). As participants might have multiple hospital stays during the study period, we used robust standard errors to account for clustering. In a sensitivity analysis, we added an interaction term between the treatment strategy and severity of mental health at entry (HoNOS score or item 1 of the HoNOS at baseline). All analyses were performed with Stata 17.

## Results

There was a total of *n* = 1219 hospitalizations during the study period. Nineteen participants were excluded because they had missing values on the HoNOS at hospital admission (1.6%), which left a final sample of *n* = 1200. At hospital discharge, 1164 participants had a completed HoNOS (retention rate = 97.0%). There were no other missing values.

Descriptive statistics and comparisons between groups are reported in Table 2. A total of 24.2% of the participants were secluded at least once during their hospital stay. Secluded participants were significantly older (*p* = .001), more likely to have an unvoluntary admission (*p* < .001), to be hospitalized in a geriatric psychiatric ward (*p* = .001), to be hospitalized for 3 weeks or more, (*p* < .001) to have higher HoNOS score (total score and item 1) at admission and discharge (*p* < .001), and to have schizophrenia, bipolar disorder or personality disorders than non-secluded participants (*p* = .03). They also had higher HoNOS scores at discharge (total score: *p* = .001, item 1: *p* < .001).

**Table 2.** Descriptive Characteristics of the Sample and Comparisons Between Groups (*n* = 1200)

	Overall *n* = 1200	Secluded *n* = 290	Non Secluded *n* = 912	OR^a^	p^a^
Age	47.9 (20.9)	51.9 (21.6)	46.7 (20.6)	1.02	.001
Gender					
Men	48.4 (581)	48.6 (141)	48.4 (440)	Ref	-
Women	51.6 (619)	51.4 (149)	51.6 (470)	0.97	.88
Nationality					
Swiss	68.0 (816)	70.0 (203)	67.4 (613)	Ref	-
Other	32.0 (384)	30.0 (87)	32.6 (297)	1.15	.53
Civil status					
Married or registered partnership	19.7 (236)	20.3 (59)	20.3 (177)	Ref	
Single, divorced, widower	80.3 (964)	79.7 (231)	79.7 (733)	1.11	.67
Previous hospitalizations in psychiatry	68.9 (827)	70.3 (204)	68.5 (623)	1.18	.44
Unvoluntary admission	52.0 (624)	79.3 (230)	43.3 (394)	6.95	<.001
Psychiatric ward					
Adult	74.5 (894)	66.2 (192)	77.1 (702)	Ref	-
Geriatrics	25.5 (306)	33.8 (98)	22.9 (208)	2.25	.001
Duration of hospitalization					
Less than 3 weeks	54.8 (657)	35.2 (102)	60.9 (554)	Ref	-
3 weeks or more	45.2 (543)	64.8 (187)	39.1 (356)	4.27	<.001
Primary psychiatric disorder					
Other disorders	40.2 (482)	35.4 (103)	41.8 (380)	Ref	-
Schizophrenia, bipolar disorder, personality disorders	59.8 (718)	64.6 (188)	58.2 (530)	1.60	.03
HoNOS At admission	24.6 (6.7)	26.7 (6.1)	24.0 (6.8)	1.08	<.001
Item 1 HoNOS at admission	2.3 (1.4)	3.0 (1.0)	2.1 (1.4)	2.16	<.001
HoNOS At discharge (0–48) (*n* = 1164)	13.2 (6.3)	14.5 (6.6)	12.8 (6.1)	1.05	.001
Item 1 HoNOS at discharge (0–4) (*n* = 1164)	0.7 (1.0)	0.9 (1.1)	0.6 (0.9)	1.56	<.001

*Note*. HoNOS: Health of the Nations Outcome Scales, OR: odd-ratio.

^a^Simple mixed-effect logistic regressions with the groups (secluded/non secluded) as the outcome variable.

Results for the primary outcome (total score of the HoNOS) and secondary outcome (item 1 of the HoNOS) are reported in Table 3. Using IP weighting to account for confounding and attrition and controlling for baseline covariates, there were significant effects of seclusion on both outcomes. Participants who were secluded had a higher HoNOS score at discharge (1.49 point, 95% confidence interval [CI]: 0.56; 2.41, *p* = .002) compared to those who were not secluded. Participants who were secluded also had a higher score on the item 1 of the HoNOS at discharge (0.25, 95% CI: 0.05; 0.45, *p* = .01) compared to those who were not secluded.

**Table 3.** Estimation of the Effect of Seclusion on the HoNOS Score (*n* = 1164)

	Outcome: HoNOS Score			Outcome: Item 1 HoNOS		
	Coefficient	*p*	95% CI	Coefficient	*p*	95% CI
Seclusion (ref. No)	1.49	.002	0.56; 2.41	0.25	.01	0.05; 0.45
Age	−0.01	.86	−0.04; 0.03	−0.01	.12	−0.01; 0.00
Gender (ref. Women)	0.73	.06	−0.03; 1.49	−0.12	.21	−0.30; 0.06
Nationality (ref. other than CH)	0.01	.98	−0.78; 0.80	0.05	.63	−0.15; 0.24
Civil status (ref. Single, divorced, widower)	−0.39	.42	−1.32; 0.54	−0.09	.44	−0.32; 0.14
Previous hospitalizations in psychiatry	1.48	.001	0.65; 2.32	0.24	.03	0.03; 0.45
Unvoluntary admission	−0.16	.70	−0.96; 0.65	0.14	.16	−0.05; 0.33
Psychiatric ward (ref. Adult)	0.23	.78	−1.33; 1.78	0.39	.04	0.02; 0.77
Duration of hospitalization (ref. Less than 3 weeks)	−0.57	.15	−1.32; 0.19	−0.10	.29	−0.28; 0.09
Primary psychiatric disorder (ref. other disorders)	−0.89	.03	−1.69; −0.10	0.01	.94	−0.19; 0.21
HoNOS at admission	0.40	<.001	0.33; 0.47	0.01	.06	−0.00; 0.03
Item 1 HoNOS at admission	−0.39	.01	−0.70; −0.09	0.29	<.001	0.20; 0.38

*Note*. Linear regression model predicting the outcome at discharge with the treatment strategy (being or not secluded), controlling for covariates, using inverse probability weighting for confounding and attrition bias, and robust standard errors.

HoNOS: Health of the Nations Outcome Scales, CI: confidence intervals.

In the sensitivity analyses, the interaction terms were not significant (HoNOS score: *p* = .351, item 1 of the HoNOS: *p* = .693, see Supplementary Table 1).

## Discussion

This study used an emulated trial to test the effect of seclusion on mental health status, assessed with the HoNOS. The total HoNOS score at discharge was used as the primary outcome and the item 1 of the HoNOS (focusing on focusing on overactive, aggressive, disruptive, or agitated behavior) was used as the secondary outcome.

The main results showed that participants who were secluded during their hospital stay had the worst mental health status when they entered the hospital and when they left. At discharge, the total HoNOS and item 1 scores were respectively 1.49 and 0.25 points higher in the secluded group compared to the non-secluded group, controlling for the confounding and attrition biases with IP weighting. The model controlled for all baseline covariate, including the HoNOS score. Thus, although seclusion was likely targeting the most severely ill and aggressive patients, this intervention did not seem helpful in reducing the burden of symptoms. These results confirm previous studies’ findings, which suggested a negative effect of coercive measures on mental health (Chieze et al., 2019; Kersting et al., 2019). Importantly, our study overcame previous methodological gaps, as it relied on a large sample size, a longitudinal design, and robust statistical methods controlling for the most important biases (Chieze et al., 2019).

However, even if the effect of the seclusion on mental health status was statistically significant, it was of small magnitude. Indeed, the HoNOS ranges from 0 to 48 points and item 1 from 0 to 4, which means that differences between groups were small. There is no established threshold regarding the clinical significance of HoNOS changes. Some authors have argued that an 8-point change might be considered as clinically relevant, while others argued for the use of a categorical approach, or a combination of both (Lay et al., 2021; Ronk et al., 2016). Of note, this 8-point change deals with an intra-individual change and not a between-group comparison, as performed in this study. There is also a debate as to the validity of the HoNOS as a unidimensional model capturing changes in mental health state. In our case, it is thus most probable that aspects related to the social and housing conditions of the patients have only been marginally improved during hospital stay. The negative effect of seclusion on mental health may effectively be small, but other reasons could explain this small magnitude. One reason was that we only assessed the presence or absence of seclusion, and not the number of seclusion episodes or the duration of seclusion. We therefore might have missed information on the intensity of seclusion, which may have an impact on patients’ mental health status. However, even if the observed changes in total HoNOS and item 1 scores might be considered as clinically marginal, the fact that seclusion has a negative impact on patients’ mental state should raise concerns.

### Clinical Implications

Important implications for clinical practice can be drawn from this study. There is a need to inform about potential negative effects of seclusion on mental health, to raise awareness about its potential adverse consequences, and to develop alternative strategies.

A therapeutic effect of coercive measures is sometimes expected by medical or nursing staff (Chieze et al., 2019; Doedens et al., 2019; Van Der Merwe et al., 2013). This therapeutic effect was not observed in our study. On the contrary, there was a negative effect, with seclusion leading to increased aggressive and disruptive behaviors, as assessed with the item 1 of the HoNOS. As aggressive behaviors’ management is an objective of the use of seclusion (Newton-Howes, 2013), our results suggest that it can be counterproductive.

We recommend a reduce the use of coercive practices and to strengthen alternative strategies, such as shared decision-making, environmental interventions, post-coercion review, de-escalation techniques, integration of peer workers, integrated care, and staff training (Barbui et al., 2021; Gooding et al., 2020; Hirsch & Steinert, 2019). We believe that a paradigm change is needed in psychiatric care.

### Limitations

This study had some limitations. First, as mentioned above, seclusion was coded as present or absent during the hospital stay. Information on seclusion were not standardized in medical files and we therefore missed reliable information on the frequency and duration of seclusion. Other types of coercive measures were rare and were not analyzed. A larger range of coercive measures should be included in further studies, along with indications of frequency and duration over the hospital stay to provide a better overview of the impact of coercive measures on mental health.

Second, we could not exclude that some unmeasured confounding variables might have affect results, even if the most important predictors of the use of coercive measures were included to derive IP weights (Beames & Onwumere, 2022; Chieze et al., 2021a, 2021b). For example, we only controlled for baseline covariates. Some measures during hospitalization and prior to seclusion would have been useful to better control for confounding. Other measures related to mental health and behavior would have been useful, as those who were more severely ill at baseline were more likely to be secluded.

Third, there is a high variability between settings in the use of coercive measures (Flammer et al., 2022). Our monocentric study’s findings may not be generalizable to other settings.

Fourth, the study took place during the SARS-CoV-2 pandemic, including periods of lockdown. The use of seclusion might have increased compared to the pre-pandemic period and findings should be interpreted in light of this context.

### Conclusion

Overall, our findings confirmed that coercive measures such as seclusion had a negative effect of patients’ mental health status, using an emulated trial that allowed causal inference. Seclusion should therefore be avoided in mental health care settings and training efforts should raise the awareness of the medical staff about potential adverse effects instead of therapeutic benefits.

## Supplemental Material

Supplemental Material - Effect of Seclusion on Mental Health Status in Hospitalized Psychiatric Populations: A Trial Emulation using Observational Data Click here for additional data file. Supplemental Material for Effect of Seclusion on Mental Health Status in Hospitalized Psychiatric Populations: A Trial Emulation using Observational Data by Stéphanie Baggio, Stefan Kaiser and Alexandre Wullschleger in Evaluation & the Health Professions
