# Supplementary material for: Effect of Seclusion on Mental Health Status in Hospitalized Psychiatric Populations: A Trial Emulation using Observational Data
Source: Eval Health Prof. 2023 Mar 10;47(1):3–10. doi: 10.1177/01632787231164489 (PMC10858627; doi:10.1177/01632787231164489)
Supplement: Supplemental Material - Effect of Seclusion on Mental Health Status in Hospitalized Psychiatric Populations: A Trial Emulation using Observational Data [file sj-pdf-1-ehp-10.1177_01632787231164489.pdf]

Supplementary Table 1. Estimation of the effect of seclusion on the HoNOS score with an interaction term between seclusion and mental health status at baseline (n=1,164)

<!--Col Count:7-->	Outcome: HoNOS score			Outcome: item 1 HoNOS		
	Coefficient	p	95% CI	Coefficient	p	95% CI
Seclusion (ref. no)	0.12	<.001	0.06; 0.18	0.23	.062	-0.01; 0.48
Age	>-0.01	.552	-0.00; 0.00	-0.01	.122	-0.01; 0.00
Gender (ref. women)	0.05	.063	-0.00; 0.00	-0.12	.202	-0.30; 0.06
Nationality (ref. other than CH)	0.01	.833	-0.05; 0.06	0.05	.622	-0.14; 0.24
Civil status (ref. single, divorced, widower)	-0.04	.323	-0.11; 0.04	-0.10	.424	-0.33; 0.14
Previous hospitalizations in psychiatry	0.11	.001	0.05; 0.17	0.24	.024	0.03; 0.46
Unvoluntary admission	-0.01	.813	-0.07; 0.05	0.14	.157	-0.05; 0.33
Psychiatric ward (ref. adult)	0.03	.561	-0.08; 0.15	0.39	.044	0.01; 0.77
Duration of hospitalization (ref. less than 3 weeks)	-0.03	.256	-0.09; 0.02	-0.10	.301	-0.28; 0.09
Primary psychiatric disorder (ref. other disorders)	-0.06	.032	-0.12; -0.01	0.00	.970	-0.19; 0.20
HoNOS at admission	0.03	<.001	0.03; 0.04	0.01	.055	-0.00; 0.03
Item 1 HoNOS at admission	-0.03	.004	-0.06; -0.01	0.28	<.001	0.19; 0.37
Interaction term[1]	-0.01	.351	-0.02; 0.01	0.04	.693	-0.16 ; 0.24

HoNOS: Health of the Nations Outcome Scales, CI: confidence intervals.

Linear regression model predicting the outcome at discharge with the treatment strategy (being or not secluded), controlling for covariates, using inverse probability weighting for confounding and attrition bias, and robust standard errors.

[1] Interaction term between seclusion and HoNOS score at admission (outcome HoNOS score) or between seclusion and item 1 HoNOS at admission (outcome item 1 HoNOS).

Field Code Changed
